# In search of preventive strategies: novel high-CBD *Cannabis sativa* extracts modulate ACE2 expression in COVID-19 gateway tissues

**DOI:** 10.18632/aging.202225

**Published:** 2020-11-22

**Authors:** Bo Wang, Anna Kovalchuk, Dongping Li, Rocio Rodriguez-Juarez, Yaroslav Ilnytskyy, Igor Kovalchuk, Olga Kovalchuk

**Affiliations:** 1 Pathway Rx Inc., Lethbridge, AB T1K 7X8, Canada; 2 Swysh Inc., Lethbridge, AB T1K 7X8, Canada; 3 University of Lethbridge, Lethbridge, AB T1K3M4, Canada; 4 University of Calgary, Cumming School of Medicine, Calgary, AB T2N 1N4, Canada

**Keywords:** COVID-19, SARS-CoV2, ACE2 receptor, medical cannabis, CBD

## Abstract

With the current COVID-19 pandemic, caused by the severe acute respiratory syndrome coronavirus 2 (SARS-CoV-2), there is an urgent need for new therapies and prevention strategies that can help curtail disease spread and reduce mortality. The inhibition of viral entry and thus spread is a plausible therapeutic avenue.

SARS-CoV-2 uses receptor-mediated entry into a human host via the angiotensin-converting enzyme 2 (ACE2), which is expressed in lung tissue as well as the oral and nasal mucosa, kidney, testes and gastrointestinal tract. The modulation of ACE2 levels in these gateway tissues may be an effective strategy for decreasing disease susceptibility.

*Cannabis sativa*, especially those high in the anti-inflammatory cannabinoid cannabidiol (CBD), has been found to alter gene expression and inflammation and harbour anti-cancer and anti-inflammatory properties. However, its effects on ACE2 expression remain unknown.

Working under a Health Canada research license, we developed over 800 new *C. sativa* cultivars and hypothesized that high-CBD *C. sativa* extracts may be used to down-regulate ACE2 expression in target COVID-19 tissues. Using artificial 3D human models of oral, airway and intestinal tissues, we identified 13 high-CBD *C. sativa* extracts that decrease ACE2 protein levels. Some *C. sativa* extracts down-regulate serine protease TMPRSS2, another critical protein required for SARS-CoV-2 entry into host cells.

While our most effective extracts require further large-scale validation, our study is important for future analyses of the effects of medical cannabis on COVID-19. The extracts of our most successful novel high-CBD *C. sativa* lines, pending further investigation, may become a useful and safe addition to the prevention/treatment of COVID-19 as an adjunct therapy.

## INTRODUCTION

There is a global pandemic of the COVID-19 disease, which is caused by the zoonotic severe acute respiratory syndrome coronavirus 2 (SARS-CoV-2). By mid-August 2020, it had spread throughout the world, affecting over 50 million people and killing over 1,250,000. Worldwide, death rates vary between 2 and 10 percent [1, 2].

SARS-CoV-2 exhibits fast human–human transmission, with a doubling time of around six to seven days and an R_o_ of around 2.2 [3, 4]. Similar to other respiratory pathogens, SARS-CoV-2 is transmitted through respiratory droplets from coughing and sneezing. However, aerosol transmission and close-contact transmission cannot be ruled out as means of disease spread [4].

The many symptoms of COVID-19 can be classified into several categories: typical influenza-like symptoms, such as fever, fatigue, myalgia and headache; respiratory symptoms, such as dry cough and dyspnea; and gastrointestinal symptoms, such as diarrhea and nausea [5]. Anosmia and ageusia, the losses of the senses of smell and taste, respectively, are also common. Overall, COVID-19 has a broad clinical spectrum ranging from asymptomatic and mild disease to pneumonia that often progresses to respiratory failure, major organ failure and death [4, 6]. Up to 20% of cases are severe and require hospital admission. Currently, there is no vaccine or any known approved drug therapy for this virus.

SARS-CoV-2 was first isolated from human airway epithelial cells [7] and found to be similar to the severe acute respiratory syndrome coronavirus (SARS-CoV) [8]. The angiotensin-converting enzyme 2 (ACE2) is the cell receptor of SARS-CoV-2 and the main route for receptor-mediated entry of the virus into human hosts [9]. Since ACE2 plays a pivotal role in cellular entry, ACE2-expressing cells serve as critical viral gateways [10].

To date, ACE2 expression has been found in lung tissue, nasal mucosa, kidney, testes and the gastrointestinal tract. High levels of ACE2 were seen in lung and intestinal epithelia [11]. An in-depth analysis of The Cancer Genome Atlas (TCGA) and Functional Annotation of The Mammalian Genome Cap Analysis of Gene Expression (FANTOM5 CAGE) datasets revealed that ACE2 is expressed in oral mucosa and enriched in the epithelial cells of the tongue. ACE2 expression in oral, lung and intestinal epithelia may thus constitute important routes of SARS-CoV-2 entry into hosts [12].

A recent study by Xu et al. reported high levels of ACE2 expression in oral epithelial tissues and suggested that the oral cavity could be highly susceptible to SARS-CoV-2 infection and thus an important target for prevention strategies [12]. Similarly, numerous studies have reported high levels of ACE2 in the lower respiratory tract; higher levels of ACE2 expression, such as those seen in smokers and patients with chronic obstructive pulmonary disease (COPD), are associated with higher COVID-19 predisposition and enhanced disease severity [13]. A recent integrated meta-analysis of scRNASeq data from 282 nasal, airway and lung parenchyma samples from 164 donors spanning fetal, child, adult and elderly groups showed that ACE2 expression in airway epithelia increases with age and is higher in men and in smoking individuals [14], suggesting a possible link to higher mortality rates.

The down-regulation of ACE2 levels in gateway tissues may thus be a plausible strategy for decreasing disease susceptibility. These strategies should be accessible, easy to use, and classified as generally recognized as safe (GRAS).

*Cannabis sativa*, especially those high in cannabidiol (CBD), has been found to affect gene expression and inflammation and is under investigation for several potential therapeutic applications against cancer and various inflammatory diseases [15, 16].

Working under a Health Canada research license, we developed over 800 new *C. sativa* cultivars and extracts. We also created a method of using them as a means to regulate the gene expression and molecular cascades that drive inflammation and other vital cellular processes (PCT/IL2019/051340;US16/711,647;PCT/IL2019/051342;US16/713,029;PCT/IL2019/051341;US16/711,655;PCT/IL2019/051343; US16/713,030). Using artificial 3D human tissue models, we show that high-CBD *C. sativa* extracts may down-regulate ACE2 expression in target COVID-19 tissues, suggesting an importance of these extracts in COVID-19 prevention.

## RESULTS

**Figure 1:**
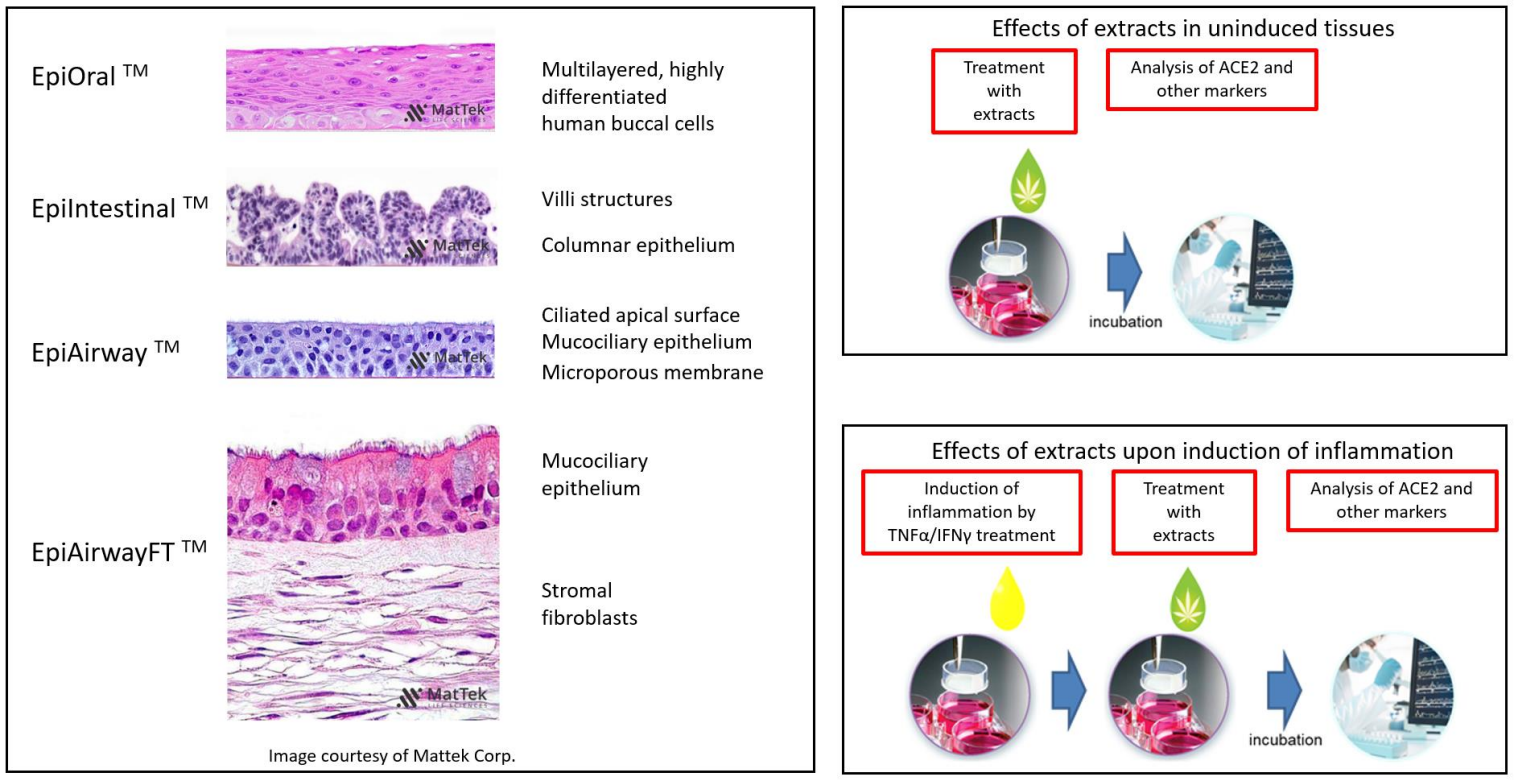
**Overview of the experimental models and setups.** H&E-stained cross sections are a courtesy of MatTek Life Sciences.

Because artificial 3D human tissue models of oral, airway and intestinal tissues are well-established and widely accepted for pathophysiology, toxicology, inflammation, virus infection and drug development studies, we used such models to analyze the effects of 23 extracts of novel *C. sativa* cultivars (#1, #5, #7, #9, #10, #31, #45, #49, #81, #90, #114, #115, #129, #130, #131, #155, #157, #166, #167, #169, #207, #274, #317) on ACE2 expression. Representative H&E stains of 3D tissue cross sections (courtesy of MatTek) are provided in Figure 1. The cannabinoid concentrations (THC, CBD, CBGA, CBN) in the flowers and extracts used in this study, as percentages of total dry weight, and the molar concentrations of the extracts are shown in Table 1.

**Table 1:** Level of single and total cannabinoids in flowers and extracts of studied *C. sativa* cultivars.

**Flowers, %**	**THC**	**CBD**	**CBGA**	**CBN**	**TOTAL Cannabinoids**	**CBD:THC ratio**
**#1**	0.25	6.79	0.12		7.16	27.16
**#5**	0.27	8.46	0		8.73	31.33
**#7**	0.21	7.2	0		7.41	34.29
**#9**	0.22	6.91	0.31		7.44	31.41
**#10**	0.53	5.54	0.42		6.49	10.45
**#31**	3.54	6.9			10.44	1.95
**#45**	0.03	1.61			1.64	53.67
**#49**	0.07	3.05			3.12	43.57
**#81**	0.46	11.81			12.27	25.67
**#90**	1.05	4.58		0.24	5.63	4.36
**#114**	0.18	6.92			7.1	38.44
**#115**	0.3	9.54			9.84	31.80
**#129**	0.28	6.75	0.66		7.69	24.11
**#130**	0.86	2.63	0.31	0.03	3.83	3.06
**#131**	0.44	6.1	0.11		6.65	13.86
**#155**	0.22	4.59	0.19	0.19	5	20.86
**#157**	0.2	3.75	0.09	0.15	4.19	18.75
**#166**	0.1	2.49	0.14		2.73	24.90
**#167**	0.08	2.25	0.16		2.49	28.13
**#169**	0.2	1.88	0.14		2.22	9.40
**#207**	8.06	7.19	0.18	0.04	15.43	0.89
**#274**	0.44	9.02	0.31		9.77	20.50
**#317**	1.13	16.4	0.68	0.1	18.31	14.50
**Extracts, %**	**THC**	**CBD**	**CBGA**	**CBN**	**TOTAL Cannabinoids**	**CBD:THC ratio**
**#1**	0.88	34.6	0.25	0.12	35.85	39.32
**#5**	1.2	35.9	0.12	0.1	37.32	29.92
**#7**	1.1	32.9	0.27	0.15	34.27	29.91
**#9**	0.98	32.6	0.97	0.15	34.55	33.27
**#10**	1.2	29.6	0.76	0.18	31.74	24.67
**#31**	19.52	27.98	0.28	0.15	47.93	1.43
**#45**	0.44	24.92	0.13	0.14	25.63	56.64
**#49**	0.28	28.48	0.23	0.11	29.1	101.71
**#81**	1.98	42.74	0.33	0.2	45.25	21.59
**#90**	4.87	19.42	0.18	0.56	25.03	3.99
**#114**	0.56	32.74	0.34	0.21	33.85	58.46
**#115**	1.23	42.52	0.42	0.28	44.45	34.57
**#129**	1.3	35.3	1.2	0.42	38.22	27.15
**#130**	2.43	28.43	0.98	0.18	32.02	11.70
**#131**	0.84	34.9	0.84	0.1	36.68	41.55
**#155**	0.45	32.1	0.56	0.25	33.11	73.57
**#157**	0.62	33.5	0.73	0.33	34.85	54.03
**#166**	0.47	34.6	0.62	0.1	35.69	73.62
**#167**	0.38	24.3	0.29	0.12	24.97	63.95
**#169**	0.67	19.28	0.45	0.18	20.58	28.78
**#207**	28.08	24.64	0.74	0.26	53.72	0.88
**#274**	0.93	43.81	1.2	0.12	46.06	47.11
**#317**	3.13	68.6	0.62	1.97	0.25	21.92
**Molarity/μM**	**THC**	**CBD**	**CBGA**	**CBN**	**TOTAL Cannabinoids**	**TOTAL Cannabinoids**
**#1**	0.28	11.00	0.08	0.04		
**#5**	0.38	11.42	0.04	0.03		
**#7**	0.35	10.46	0.09	0.05		
**#9**	0.31	10.37	0.31	0.05		
**#10**	0.38	9.41	0.24	0.06		
**#31**	6.21	8.90	0.09	0.05		
**#45**	0.14	7.92	0.04	0.05		
**#49**	0.09	9.06	0.07	0.04		
**#81**	0.63	13.59	0.10	0.06		
**#90**	1.55	6.18	0.06	0.18		
**#114**	0.18	10.41	0.11	0.07		
**#115**	0.39	13.52	0.13	0.09		
**#129**	0.41	11.23	0.38	0.14		
**#130**	0.77	9.04	0.31	0.06		
**#131**	0.27	11.10	0.27	0.03		
**#155**	0.14	10.21	0.18	0.08		
**#157**	0.20	10.65	0.23	0.11		
**#166**	0.15	11.00	0.20	0.03		
**#167**	0.12	7.73	0.09	0.04		
**#169**	0.21	6.13	0.14	0.06		
**#207**	8.93	7.84	0.23	0.08		
**#274**	0.30	13.93	0.38	0.04		
**#317**	1.00	21.81	0.62	0.08		

### Cannabis extracts regulate ACE2 expression in normal/unstimulated 3D tissues

### Airway tissues

**Figure 2:**
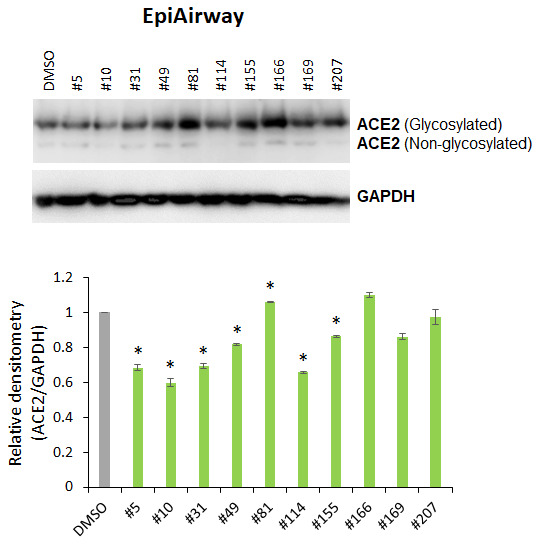
**Effects of novel *C. sativa* extracts on the levels of ACE2 in the normal uninduced human EpiAirway tissue models.** Two tissue samples were used per treatment group. Protein extracts were prepared from each sample, and equal amounts of each sample in each group were pooled together. Each bar is an average (with SD) from three technical repeat measurements. * - p<0.05, Student’s t-test.

We analyzed the effect of cannabis extracts #5, #10, #31, #49, #81, #114, #155, #166, #169 and #207 on ACE2 expression in 3D EpiAirway tissues. Western blot analysis showed that extracts #5, #10, #31, #49, #114 and #155 significantly (p<0.05) down-regulated the ACE2 expression (Figure 2), whereas extracts #81 and #166 slightly increased its expression.

### Oral tissues

**Figure 3:**
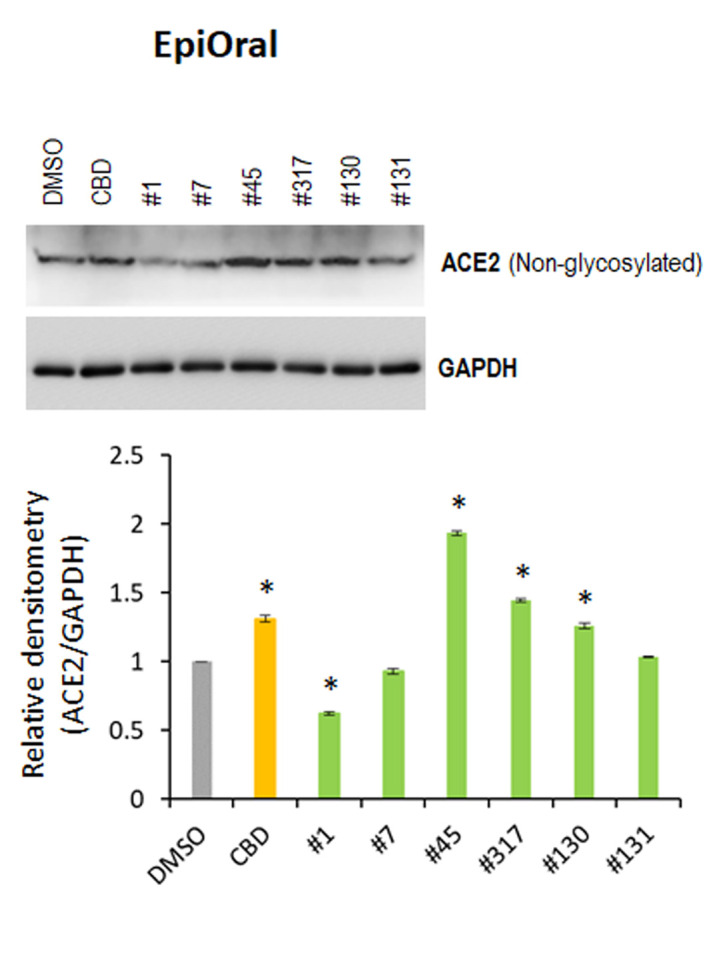
**Effects of novel *C. sativa* extracts on the levels of ACE2 in the normal uninduced EpiOral tissue models.** Three tissue samples were used per treatment group. Protein extracts were prepared from each sample, and equal amounts of each sample in each group were pooled together. Each bar is an average (with SD) from three technical repeat measurements. * - p<0.05, Student’s t-test.

We also examined the effect of extracts #1, #7, #45, #317, #130 and #131 and of CBD alone on ACE2 expression in 3D EpiOral tissues. Western blot analysis indicated that extracts #1 and #7 down-regulated ACE2 expression (Figure 3), whereas CBD and extracts #45, #317 and #130 up-regulated its expression. These results suggest that cannabis extracts may differentially regulate ACE2 expression in normal human 3D tissues.

### Cannabis extracts modulate ACE2 expression in inflammation-stimulated 3D tissues

We examined the effect of cannabis extracts on the expression of ACE2 in inflammation-stimulated 3D tissues, since inflammation is a significant component of viral disease.

### Oral tissues

**Figure 4:**
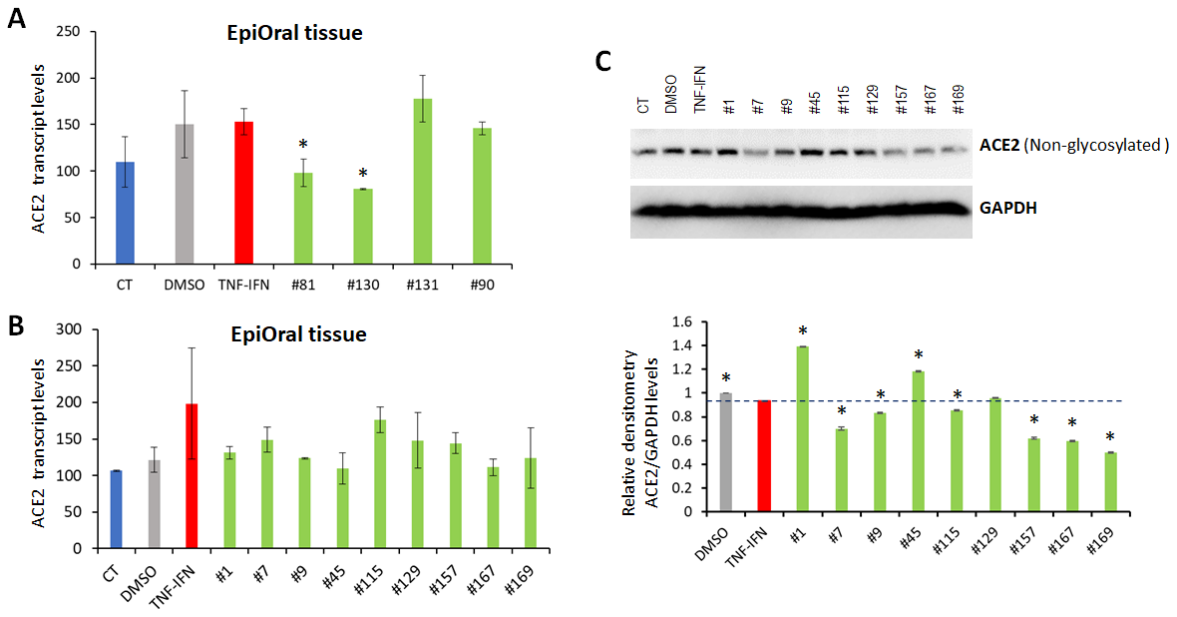
**Effects of novel *C. sativa* extracts on the levels of ACE2 in the EpiOral tissue models upon induction of inflammation by treatment with TNFα/IFNγ.** (**A**, **B**) Total RNA was isolated from 3D EpiOral tissues and subjected to RNA-Seq analysis as described in the “Methods”. The levels of ACE2 gene expression is presented as an average (with SD) from two samples. * - Statistically significant, ANOVA-like analysis and pair-wise comparison, as per Materials and Methods. (**C**) Two tissue samples were used per treatment group. Whole lysates prepared from EpiOral tissues were subjected to Western blotting using antibody against ACE2 as described in the “Methods”. The relative densitometry is presented as an average (with SD) from three technical repeat measurements. * - p<0.05, Student’s t-test.

The 3D EpiOral tissues were treated with TNFα/IFNγ for 24 h, followed by application of the indicated extracts for another 24 h. In an initial experiment, we used RNASeq to analyze the effects of four extracts (#81, #90, #130, #131) of the novel cannabis cultivars on the levels of ACE2 expression in the EpiOral tissues after inflammation induction by TNFα/IFNγ. Extracts #81 and #130 significantly down-regulated the levels of the ACE2 transcripts in the EpiOral tissues (ANOVA-like analysis, padj=2.14e-06 for both extracts; pair-wise comparison between DMSO and extract #130 and between DMSO and extract #81, padj<0.05) (Figure 4A). In a larger-scale follow-up experiment, we investigated the effect of nine extracts (#1, #7, #9, #45, #115, #129, #157, #167, #169) of the novel cannabis cultivars. The RNASeq analysis showed that TNFα/IFNγ up-regulated the ACE2 mRNA levels (Figure 4B), and all extracts except #115 down-regulated the expression of the TNFα/IFNγ-induced changes in ACE2, although not significantly. Interestingly, Western blot analysis showed that extracts #9 and #45 increased ACE2 protein levels (Figure 4C), whereas #1, #7, #115, #157, #167 and #169 down-regulated the protein levels.

### Intestinal tissues

**Figure 5:**
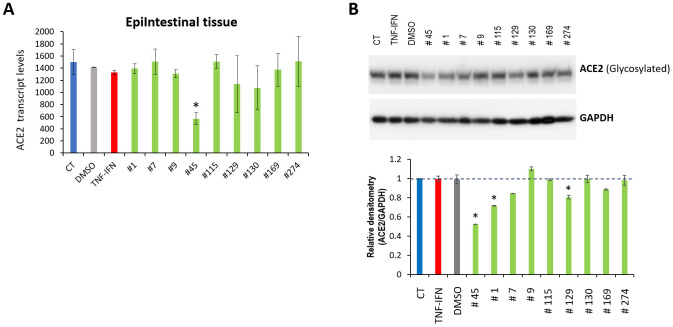
**Effects of novel *C. sativa* extracts on the levels of ACE2 in the EpiIntestinal tissue models upon induction of inflammation by treatment with TNFα/IFNγ.** (**A**) Total RNA was isolated from 3D EpiItestinal tissues and subjected to RNA-Seq analysis as described in the “Methods”. The levels of ACE2 gene expression is presented as an average (with SD) from two samples. * - Statistically significant, ANOVA-like analysis and pair-wise comparison, as per Materials and Methods. (**B**) Two tissue samples were used per treatment group. Whole lysates prepared from EpiIntestinal tissues were subjected to Western blotting using antibody against ACE2 as described in the “Methods”. The relative densitometry is presented as an average (with SD) from three technical repeat measurements. * - p<0.05, Student’s t-test.

We treated 3D EpiIntestinal tissues with TNFα/IFNγ for 24 h and then exposed the tissues to the indicated extracts for another 24 h. RNASeq analysis indicated that extract #45 significantly down-regulated the ACE2 mRNA levels (Figure 5A); extracts #129 and #130 had a tendency to decrease ACE2 expression, but the difference was not significant.

Western blot analysis showed that, in addition to extracts #45 and #129, which decreased the ACE2 protein levels (Figure 5B) and confirmed the RNASeq results, extracts #1, #7 and #169 down-regulated the ACE2 protein levels, while #9, #115, #130 and #274 had no effect.

### Airway tissues

To analyze the effects of the cannabis extracts on the ACE2 expression in the inflammation-stimulated airway tissues, we used two types of 3D tissue models: EpiAirway and EpiAirway Full Thickness (EpiAirway-FT). The full-thickness tissues contain mucociliary epithelium as well as stromal fibroblasts, unlike the EpiAirway tissues, which contain only the epithelial component (Figure 1).

**Figure 6:**
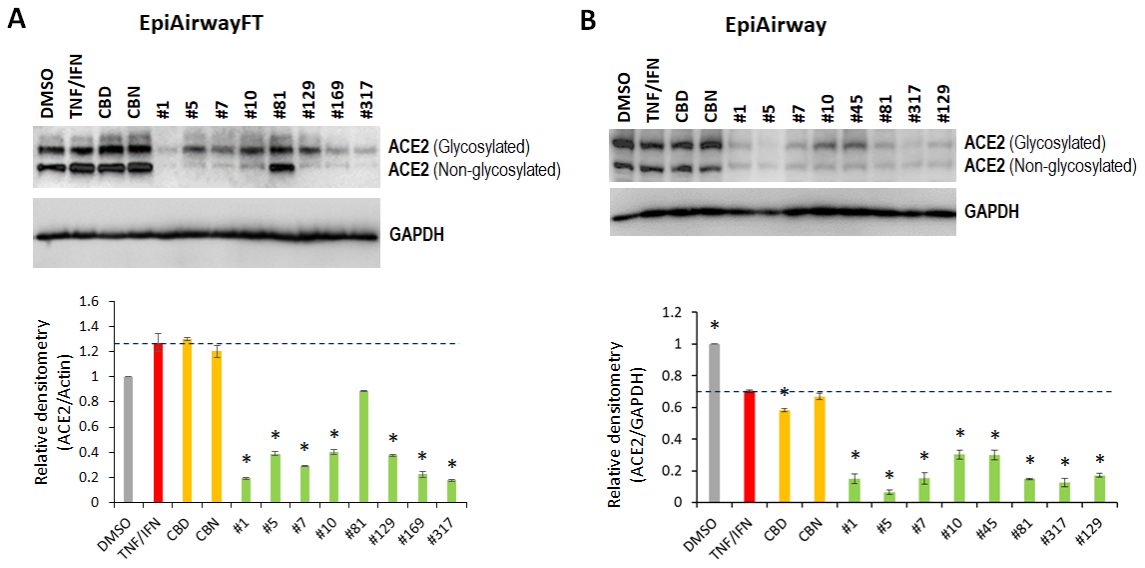
Effects of novel *C. sativa* extracts and cannabinoids on the levels of ACE2 in the EpiAirway-FT (**A**) and EpiAirway (**B**) tissue models upon induction of inflammation by treatment with TNFα/IFNγ. Three tissue samples were used per treatment group. Protein extracts were prepared from each sample, and equal amounts of each sample in each group were pooled together. Each bar is an average (with SD) from three technical repeat measurements. * - p<0.05, Student’s t-test.

To delineate the effects of the extracts on the inflammation-simulated EpiAirway-FT, we first treated the EpiAirway-FT tissues with either TNFα/IFNγ, or TNFα/IFNγ supplemented with single cannabinoids (CBD or cannabinol [CBN]) or with the indicated *C. sativa* extracts for 48 h. CBD was chosen because of its documented anti-inflammatory properties and its abundance in the studied extracts. CBN was selected as it is a non-psychoactive cannabinoid that has many properties similar to delta-9-tetrahydrocannabinol (Δ^9^-THC). Western immunoblotting showed that TNFα/IFNγ triggered an increase in the ACE2 expression in the EpiAirway-FT tissues (Figure 6A). Remarkably, all the examined extracts attenuated the TNFα/IFNγ-induced ACE2 expression, while CBD and CBN had no effect on ACE2 expression (Figure 6A). Similar results were also observed in an independent experimental repeat using the 3D EpiAirway tissues (Figure 6B). Together, these results suggest that the cannabis extracts modulated the ACE2 expression in the inflammation-stimulated 3D tissues, while CBD or CBN alone did not affect the ACE2 levels.

### Cannabis extracts affect TMPRSS2 expression

**Figure 7:**
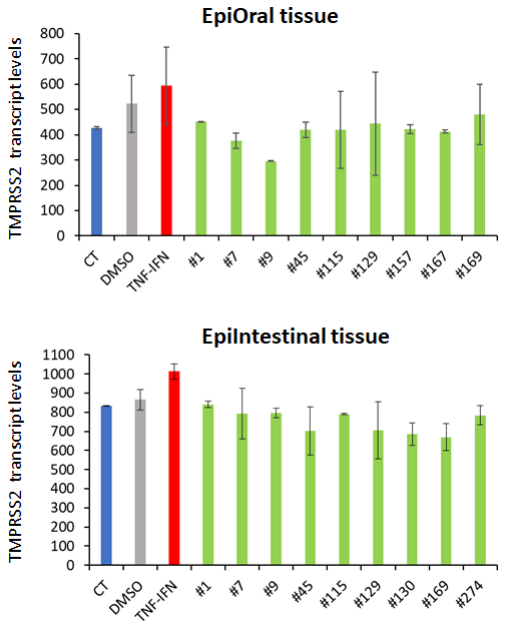
**Effects of novel *C. sativa* extracts on the levels of TMPRSS2 gene expression in the EpiOral and EpiIntestinal tissue models.** Each bar is an average (with SD) of two samples, as per Materials and Methods. The Y axis shows the arbitrary units of TMPRSS2 gene expression, while the X axis shows the samples.

Along with ACE2, the serine protease TMPRSS2 plays an important role in the SARS-CoV-2 infection process. While ACE2 is the receptor for viral entry, TMPRSS2 primes viral spike proteins and is therefore crucial for SARS-CoV-2 entry into host cells. Recent studies reveal that TMPRSS2 inhibitors block virus entry [17]. RNASeq analysis indicated that TNFα/IFNγ triggered TMPRSS2 expression, which was somewhat attenuated by all the extracts tested (Figure 7).

**Figure 8:**
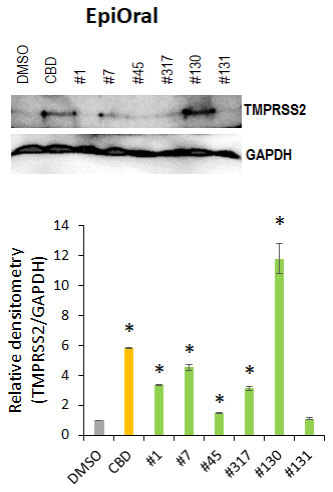
**Effects of novel *C. sativa* extracts on the levels of TMPRSS2 in the normal uninduced EpiOral tissue models.** Three tissue samples were used per treatment group. Protein extracts were prepared from each sample, and equal amounts of each sample in each group were pooled together. Each bar is an average (with SD) from three technical repeat measurements. * - p<0.05, Student’s t-test.

We further explored whether or not the cannabis extracts affected the TMPRSS2 protein levels in the oral and airway tissues. We found that application of extracts #1, #45, #317 and #131 decreased TMPRSS2 levels compared with CBD alone, which up-regulated TMPRSS2 in the oral tissues (Figure 8). Tested extracts (except #131) and CBD increased the TMPRSS2 levels compared with DMSO.

**Figure 9:**
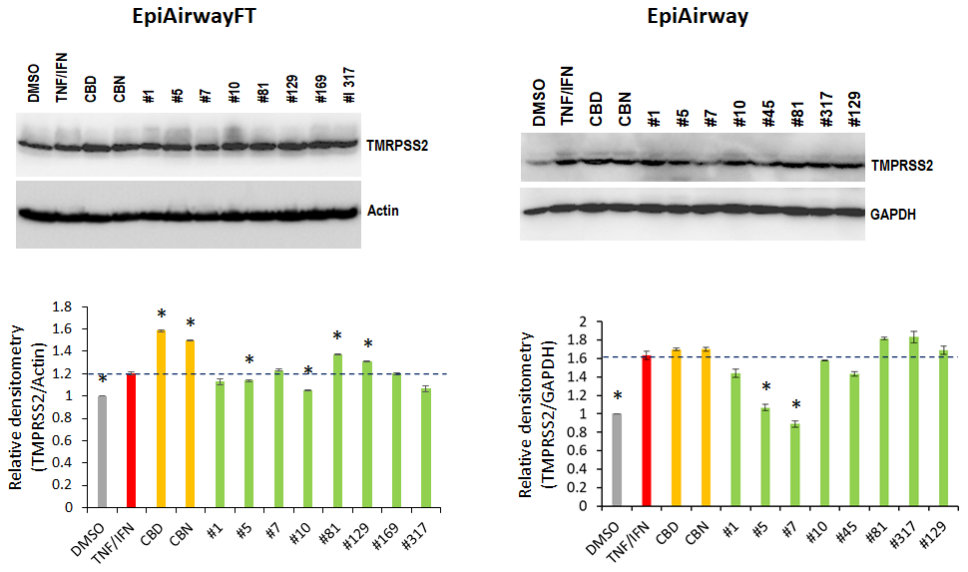
****Effects of novel *C. sativa* extracts on the levels of TMPRSS2 in the EpiAirway-FT (**A**) and EpiAirway (**B**) tissue models upon induction of inflammation by treatment with TNFα/IFNγ. Three tissue samples were used per treatment group. Protein extracts were prepared from each sample, and equal amounts of each sample in each group were pooled together. Each bar is an average (with SD) from three technical repeat measurements. * - p<0.05, Student’s t-test.

In the TNFα/IFNγ-stimulated EpiAirway-FT tissues, TNFα/IFNγ increased the TMPRSS2 levels. Application of CBD, CBN and extracts #81 and #129 increased the TMPRSS2 protein levels, while extracts #5, #10 and #317 slightly decreased them compared with TNFα/IFNγ alone (Figure 9A). In the TNFα/IFNγ-stimulated EpiAirway tissues, extracts #1, # 5, #7 and #45 down-regulated the TNF/IFN-induced TMPRSS2 expression, while extracts #10 and #129, CBD and CBN did not affect its levels (Figure 9B).

To determine whether ACE2 and TMPRSS2 inhibition was dependent on the level of a single cannabinoid, total cannabinoids or their ratio, we correlated the down-regulation level of ACE2 and TMPRSS2 with the level of cannabinoids for each experiment. Interestingly, we found a negative correlation between the down-regulation fold of ACE2 and all individual cannabinoids or total cannabinoid content in most experiments (Supplementary Table 1).

Next, we analyzed the correlation between ACE2 and TMPRSS2 effects and total terpene concentrations for several cultivars for which we had data (extracts #1, #7, #9, #81 and #317) (Table 2). We found a strong positive correlation between the efficiency of the extracts in the down-regulation of ACE2 and the total level of terpenes in these extracts in experiments analyzing the EpiOral, EpiIntenstinal and EpiAirway transcriptomes (0.99, 0.97 and 0.97, respectively, as seen in Supplementary Table 2). In contrast, the correlation between the terpenes and protein levels (Western blot) was not as evident; weak negative correlations were found for the EpiOral and EpiIntestinal tissues (−0.31 and −0.29, respectively). The correlation for TMPRSS2 was also weak.

**Table 2:** Level of total terpenes in flowers of selected *C. sativa* cultivars.

**Flowers**	**Terpenes, %**
#1	12.34
#7	6.95
#9	14.02
#81	4.55
#317	1.93

We next compared the degree of down-regulation of ACE2 in the different tissues and cells. We found that, on average, the effect was most prominent on the EpiAirway tissues. As far as the individual extracts were concerned, the strongest effect was observed for extracts #1, #129, #7 and #5 (Supplementary Table 3).

## DISCUSSION

The observed down-regulation of ACE2 gene expression by several tested extracts of new *C. sativa* cultivars is a novel and crucial finding. Our results establish a foundation for further in-depth analyses of the effects of *C. sativa* on the molecular etiology and pathogenesis of COVID-19 and other viral diseases wherein the viruses use the ACE2 receptor as a molecular gateway. If these results are further confirmed, these high-CBD cannabis extracts can be used to develop prevention strategies directed at lowering ACE2 levels in high-risk gateway tissues. ACE2 level modulation is of particular importance since it appears to change throughout disease progression, and some studies show that ACE2 is essential for lung function in animal models of SARS [18, 19]. It would also be important to test the effects of *C. sativa* lines on other receptors involved in SARS-CoV-2 entry for their anti-inflammatory potential.

Furthermore, cannabis has over 100 phytocannabinoids [20], of which the main ones are Δ^9^-THC and CBD [21]. Cannabis possesses many minor cannabinoids, such as cannabigerol (CBG) and CBN, and numerous terpenes. Terpenes are responsible for variations in scent and may act synergistically with cannabinoids, with the potential to strongly enhance cannabinoid effects. Terpenes and minor cannabinoids are responsible for the ‘entourage effect’ [21], whereby whole plant extracts have more pronounced biological effects than individual cannabinoids. Here, we did not find a strong correlation between CBD levels and the observed down-regulation of ACE2 and TMPRSS2. Hence, we may indeed be observing the entourage effect in action, and the effects of extracts on ACE2 expression may not necessarily be attributed to CBD. In the future, it would be important to identify the cannabinoids and terpenes responsible for these observed effects, although, based on the entourage effect, one could predict that whole flower extracts may be more potent than single compounds [22, 23]. Here, we noted that the effects of extracts were more pronounced than those of CBD or CBN that alone did not affect ACE2 levels (Figure 6). In the future, it would be important to undertake further studies of other isolate cannabinoids and terpenes and determine their biological effects in well-defined conditions, as well as conduct reconstruction experiment to identify the key active ingredients or combinations.

Moreover, a notable aspect of our study is that, while the molar amounts and ratios of the major cannabinoids (THC and CBD) were similar between the analyzed extracts (Table 1), not all extracts were equally effective. Some produced undesired molecular effects on the levels of the ACE2 gene and protein. This finding emphasizes that different medical cannabis cultivar may have different effects, and each cannabis cultivar has to be analyzed in detail to identify the ones that are the most potent.

Another intriguing observation is the potential tissue-specificity of cannabis effects. Interestingly, extract #45 upregulated ACE2 expression in EpiOral tissues, but down-regulated it in the EpiIntestinal and EpiAirway tissues. Overall, tissue specificity of cannabis effects needs to be further explored in detail.

Within this study, extracts were applied via media to model medical delivery, such as local oral cavity applications, encapsulated extracts and dosed oils, and inhalers or nebulizers. Therefore, our results cannot be extrapolated to the effects of cannabis smoking. Moreover, in light of recent findings showing that tobacco smoking increases ACE2 levels and exacerbates the clinical outcomes of COVID-19 [24], the effects of cannabis smoking on the levels of ACE2 expression should be carefully investigated. Interestingly, animal model studies have shown that CBD decreased inflammation and improved lung function in murine models of acute lung injury [25]. In addition, a recent article suggested that CBD can be a useful addition to current COVID-19 protocols [25].

Most importantly, seven active cultivars have less than 0.3% total THC and therefore can be classified as hemp CBD in Canada and the USA, allowing for easy implementation.

However, our study has limitations. Our initial experiments were conducted using two replicates per condition. While it is feasible to conduct a differential expression study with only two replicates, the capability to detect true differentially expressed genes (DEGs) is reduced. Indeed, a recent study by Lamarre et al. (2018) [26] analyzed the impact of the number of replicates on the number of DEGs. For a library size of 15–20 million reads, which is consistent with that of the current study, Lamarre et al. detected 14,000 DEGs. The DEG number increased by about 1,000 with every increase in the number of replicates, and the DEG increase rate slowed down after five replicates were reached. A power analysis presented in the same study showed that, with a sequencing depth of 15–20 million reads, one would expect to detect 80%–90% DEGs with a two-replicate design, depending on the level of expression (low, medium, high). Lamarre et al.’s specificity and sensitivity analysis, which compared four different DEG detection tools (including DESeq2, which was used in the present study), showed a 40%–50% true positive rate (TPR), 0.2%–2% false-positive rate (FPR) and 0.01 FDR [26]. Moreover, Lamarre and colleagues performed their analyses using individual organisms, as opposed to the 3D tissues in our case. We expect the biological variance between 3D tissue samples to be lower compared with separate plants or animals, resulting in a further reduction of the replicate number required to reach the same statistical power [26]. However, we do acknowledge the limited statistical power of our experiment; the addition of replicates would expand the list of DEGs and enhance the ability to detect significant genes at fewer fold changes.

To substantiate our data we, therefore, conducted additional experiments using EpiAirway, EpiAirway FT, and EpiOral tissues with three replicates per group, and further confirmed the effectiveness of several novel cannabis cultivars in modulation of the ACE2 expression. Nonetheless, future studies are needed to establish the precise mechanisms of action of high-CBD cannabis extracts, their tissue specificity, and the effects on ACE2, given the new knowledge on the tissue and disease stage specificity of ACE 2 expression.

Our original experiments were designed to screen the biological activities of novel cannabis extracts in 3D human tissue models. They allowed us to pinpoint the important effects of cannabis on the levels of ACE2 and TMPRSS2 expression. Further studies are needed and being undertaken to link the levels of ACE2 and inflammation. Indeed, higher ACE2 expression after SARS-CoV-2 infection is correlated with a cytokine surge in patients [8], which is thought to occur via activation of the NLRP3 inflammasome [27]. ACE2 levels are affected by age [14], and ACE2 blockers have an anti-aging potential [28]. Furthermore, linking ACE2 levels, inflammation and aging is extremely important, as aging is an inflammation-related condition. Inflamm-aging, a certain level of chronic inflammation that develops with advanced age, increases the rate of biological aging and underlies numerous age-related diseases [29]. Inflamm-aging and macroph-aging, a related phenomenon that involves senescence-related changes in macrophages, and their contributions to COVID-19 need to be further investigated [30].

## CONCLUSIONS

While our most efficacious extracts require further validation through large-scale analyses, our study is important for future analyses of the effects of medical cannabis on COVID-19. Given the current dire and rapidly developing epidemiological situation, every possible therapeutic opportunity needs to be considered and researched.

## MATERIALS AND METHODS

### Plant growth, extract preparation

All cannabis plants were grown in the licensed facility at the University of Lethbridge (license number LIC-62AHHG0R77-2019). *C. sativa* cultivars #1, #5, #7, #9, #10, #31, #45, #49, #81, #90, #114, #115, #129, #130, #131, #155, #157, #166, #167, #169, #207, #274, #317 were used for the experiments. Four plants per cultivar were grown at 22° C 18 h light 6 h dark for 4 weeks and then transferred to the chambers with 12 h light/12 h dark regime to promote flowering. Plants were grown to maturity and flowers were harvested and dried. Flower samples from four plants per cultivar were combined and used for extraction. Three grams of the powdered plant tissue per each line were used for extraction. Plant material was placed inside a 250 mL Erlenmeyer flask, 100 mL of Ethyl Acetate was poured into each flask. The flasks were covered with tin foil and incubated overnight in the dark at 21C with continuous shaking at 120 rpm. Extracts were filtered, concentrated using a rotary vacuum evaporator and transferred to a tared 3-dram vial. The leftover solvent was evaporated to dryness in an oven overnight at 50° C to eliminate the solvent completely. Levels of cannabinoids was analysed using Agilent Technologies 1200 Series HPLC system. The extract stocks were prepared from the crude extracts whereby 3-6 mg of crude extract were dissolved in DMSO (Dimethyl sulfoxide anhydrous, Life Technologies) to reach 60 mg/mL final concentration and stored at -20° C. Appropriate cell culture media (RPMI + 10% FBS or EMEM + 10% FBS) were used to dilute the 60 mg/mL stock to make working medium containing 0.01 mg/mL. Extracts were sterilized using 0.22 μm filter. Extract composition are shown in Table 1.

### Tissue models and treatments

### Tissue models

EpiAirway^TM^, EpiAirwayFT^TM^, EpiOral^TM^, EpiIntestinal^TM^ tissues were purchased from Mattek Life Sciences (Ashland, MA), equilibrated for 24 h and cultured according to manufacturer’s instructions.

### EpiAirway tissues (AIR-100)

Mattek’s EpiAirway tissue model is a human 3D mucociliary tissue model that consists of normal, human-derived tracheal/bronchial epithelial cells, is cultured at the air-liquid interface and fully recapitulates the barrier, mucociliary responses, infection, toxicity responses of human airway tissues *in vivo* (Mattek Life Sciences, MA). Two tissues were used per extract in experiments shown in Figure 3. Three tissues were used per extract in experiments shown in Figures 6B, 9B.

### EpiOral tissues (ORL-200)

MatTek’s EpiOral tissues consist of normal, human-derived oral epithelial cells. The cells have been cultured to form multilayered, highly differentiated models of the human buccal (EpiOral) phenotypes. The tissues are cultured on specially prepared cell culture inserts using serum free medium and attain levels of differentiation on the cutting edge of in vitro cell culture technology. The EpiOral tissue models exhibit in vivo-like morphological and growth characteristics which are uniform and highly reproducible (Mattek Life Sciences, MA). Two tissues were used per extract in experiments shown in Figures 4, 7. Three tissues were used per extract in experiments shown in Figures 3, 8.

### EpiIntestinal tissues (SMI-100)

EpiIntestinal tissues are 3D highly differentiated tissue models produced from normal, human cell-derived small intestine epithelial and endothelial cells and fibroblasts. Grown at the air-liquid interface, EpiIntestinal tissue models are similar to in vivo human epithelial tissues and exhibit columnar shaped basal cells and Kerckring folds, as well as brush borders, functional tight junctions and mucous secreting granules (Mattek Life Sciences, MA). Two tissues were used per extract in experiments shown in Figures 5, 7.

### EpiAirwayFT (AFT-100)

EpiAirwayFT is a ready-to-use, 3D mucociliary tissue model consisting of normal, human-derived tracheal/bronchial epithelial cells and normal human stromal fibroblasts (Mattek Life Sciences, MA). H&E-stained section of EpiAirwayFT tissue (courtesy of Mattek) exhibits a pseudostratified epithelium with ciliated cells and an extracellular matrix containing fibroblasts on a microporous membrane (Figure 1). Three tissues were used per extract (Figures 6A, 9A).

### Treatments

### EpiAirway tissue treatment (Figure 2)

Tissues were equilibrated for 24 h. The extracts or vehicle (DMSO) were dissolved in media and applied to the media surrounding the tissues. Tissues were incubated with 0.015 μg/μL of extracts for 24 h and flash frozen for RNA and protein analysis. Two tissues were used per treatment.

### EpiOral tissue treatment (Figures 3, 8)

Tissues were equilibrated for 24 h, after equilibration tissues were treated with 10 μM CBD or 0.015 μg/μL extracts for 24 h. Three tissues were used per treatment.

### EpiIntestinal and EpiOral tissue treatments (data shown in Figures 4, 5)

Tissues were equilibrated for 24 h. Inflammation was induced by treatment with proinflammatory cytokines (10 ng/ml TNFα /IFN γ for 24 hours) and then the extracts or vehicle (DMSO) were dissolved in media and applied to the media surrounding the tissues. Tissues were further incubated with 0.015 μg/μL of extracts for 24 h and flash frozen for RNA and protein analysis. Two tissue were used per treatment.

### EpiAirway and EpiAirway FT tissue treatments (Figures 6, 7, 9)

Tissues were equilibrated for 24 h, after equilibration tissues were treated with 10 ng/mL TNFα /IFN γ alone or in combination with 5 μM CBD, 5 μM CBN, or 0.015 μg/μL extracts for 48 h. Three tissues were used per treatment.

### Gene expression analysis

### RNA extraction

Two tissues per group were used for the analysis of gene expression profiles. RNA was extracted from tissues using TRIzol® Reagent (Invitrogen, Carlsbad, CA), further purified using an RNAesy kit (Qiagen), and quantified using Nanodrop2000c (ThermoScientific). Afterwards, RNA integrity and concentration were established using 2100 BioAnalyzer (Agilent).

### Library construction and sequencing

In all cases, the sequencing libraries were prepared using NEBNext Ultra II mRNA library preparation kit for Illumina (NEB) following the manufacturer’s instructions. The samples were processed by the same technician at the same time to avoid the introduction of technical batch effects. The cDNA fragment libraries were sequenced using NextSeq500 sequencing analyzer (Illumina). The samples were balanced evenly across the lanes of the sequencing flowcell.

### Statistics and bioinformatics analysis

Base-calling and demultiplexing were done with Illumina CASAVA v.1.9 bioinformatics pipeline. The base qualities were examined using FastQC v.0.11.8. The adapters and low-quality bases were trimmed using Trim Galore! v.0.6.4 www.bioinformatics.babraham.ac.uk/projects/trim_galore/. Trimmed reads were mapped to the human genome version GRCh37 using HISAT2 version 2.0.5 [31]. Counts of reads mapping to the gene as a meta-feature were obtained using featureCounts v.1.6.1 [32] taking to account the directionality of the sequencing libraries. Counts of reads mapping to features were loaded into R v.3.6.1 and normalized using DESeq2 v.1.24.0 Bioconductor package as described in the manual [33].

Two samples were used per group. The differences between all experimental groups were examined using the likelihood ratio test (LRT) test implemented in DESeq2. The reduced model included the intercept and the full model was the experimental group (Cannabis extracts and controls). Multiple comparisons adjustment of p-values was conducted by Benjamini-Hochberg procedure [34]. Specific comparisons between groups were extracted using results() function with contrast argument specified. Genes with adjusted p-values below 0.05 were considered significant. The results of statistical tests for the ACE2 receptor (Ensembl gene identifier: ENSG00000130234) were selected from the list of significant genes.

### Western blot analysis

After treatment with cannabis extracts for the indicated time, whole cellular lysates of 3D tissues were prepared in radioimmunoprecipitation assay buffer using 2.0 mm ZR BashingBead beads (Zymo Research). tissue lysates were prepared from all samples, then equal amount of each sample from each group were pooled together. Proteins (30-100 μg per sample) were electrophoresed in 10% sodium dodecyl sulfate polyacrylamide gel and electrophoretically transferred to polyvinylidene difluoride membranes (Amersham Hybond^TM^-P, GE Healthcare) at 4° C for 1.5 h. The blots were incubated for 1 h with 5% nonfat dry milk to block nonspecific binding sites and subsequently incubated at 4° C overnight with 1:1000 dilution of polyclonal antibody against ACE2 (Abcam) or TMPRSS2 (Abcam). Immunoreactivity was detected using a peroxidase-conjugated antibody and visualized with the ECL Plus Western Blotting Detection System (GE Healthcare). The blots were stripped before reprobing with antibody against GAPDH (Santa Cruz Biotechnology). Quantification of Western blot bands was performed using ImageJ in duplicate or triplicate as technical replicates. 

### Statistics

The student’s *t* test was used to determine the statistical significance of differences between groups in ACE2 and TMPRSS2 expression. A value of *p* < 0.05 was considered statistically significant.

## Supplementary Material

Supplementary Tables

## References

[1] Gallo G, Trompetto M. The effects of COVID-19 on academic activities and surgical education in Italy. J Invest Surg. 2020; 33:687–89. 10.1080/08941939.2020.1748147 32249660

[2] Spinelli A, Pellino G. COVID-19 pandemic: perspectives on an unfolding crisis. Br J Surg. 2020; 107:785–87. 10.1002/bjs.11627 32191340 PMC7228411

[3] Zhao S, Lin Q, Ran J, Musa SS, Yang G, Wang W, Lou Y, Gao D, Yang L, He D, Wang MH. Preliminary estimation of the basic reproduction number of novel coronavirus (2019-nCoV) in China, from 2019 to 2020: A data-driven analysis in the early phase of the outbreak. Int J Infect Dis. 2020; 92:214–217. 10.1016/j.ijid.2020.01.050 32007643 PMC7110798

[4] Cascella M, Rajnik M, Cuomo A, Dulebohn SC, Di Napoli R. Features, Evaluation, and Treatment of Coronavirus. 2020. In: StatPearls [Internet]. Treasure Island (FL): StatPearls Publishing; 2020. 32150360

[5] Wang D, Hu B, Hu C, Zhu F, Liu X, Zhang J, Wang B, Xiang H, Cheng Z, Xiong Y, Zhao Y, Li Y, Wang X, Peng Z. Clinical characteristics of 138 hospitalized patients with 2019 novel coronavirus-infected pneumonia in Wuhan, China. JAMA. 2020; 323:1061–69. 10.1001/jama.2020.1585 32031570 PMC7042881

[6] Zhou P, Yang XL, Wang XG, Hu B, Zhang L, Zhang W, Si HR, Zhu Y, Li B, Huang CL, Chen HD, Chen J, Luo Y, et al. A pneumonia outbreak associated with a new coronavirus of probable bat origin. Nature. 2020; 579:270–73. 10.1038/s41586-020-2012-7 32015507 PMC7095418

[7] Zhu N, Zhang D, Wang W, Li X, Yang B, Song J, Zhao X, Huang B, Shi W, Lu R, Niu P, Zhan F, Ma X, et al, and China Novel Coronavirus Investigating and Research Team. A novel coronavirus from patients with pneumonia in China, 2019. N Engl J Med. 2020; 382:727–33. 10.1056/NEJMoa2001017 31978945 PMC7092803

[8] Lu R, Zhao X, Li J, Niu P, Yang B, Wu H, Wang W, Song H, Huang B, Zhu N, Bi Y, Ma X, Zhan F, et al. Genomic characterisation and epidemiology of 2019 novel coronavirus: implications for virus origins and receptor binding. Lancet. 2020; 395:565–74. 10.1016/S0140-6736(20)30251-8 32007145 PMC7159086

[9] Turner AJ, Hiscox JA, Hooper NM. ACE2: from vasopeptidase to SARS virus receptor. Trends Pharmacol Sci. 2004; 25:291–94. 10.1016/j.tips.2004.04.001 15165741 PMC7119032

[10] Zou X, Chen K, Zou J, Han P, Hao J, Han Z. Single-cell RNA-seq data analysis on the receptor ACE2 expression reveals the potential risk of different human organs vulnerable to 2019-nCoV infection. Front Med. 2020; 14:185–92. 10.1007/s11684-020-0754-0 32170560 PMC7088738

[11] Hamming I, Timens W, Bulthuis ML, Lely AT, Navis G, van Goor H. Tissue distribution of ACE2 protein, the functional receptor for SARS coronavirus. A first step in understanding SARS pathogenesis. J Pathol. 2004; 203:631–37. 10.1002/path.1570 15141377 PMC7167720

[12] Xu H, Zhong L, Deng J, Peng J, Dan H, Zeng X, Li T, Chen Q. High expression of ACE2 receptor of 2019-nCoV on the epithelial cells of oral mucosa. Int J Oral Sci. 2020; 12:8. 10.1038/s41368-020-0074-x 32094336 PMC7039956

[13] Leung JM, Yang CX, Tam A, Shaipanich T, Hackett TL, Singhera GK, Dorscheid DR, Sin DD. ACE-2 expression in the small airway epithelia of smokers and COPD patients: implications for COVID-19. Eur Respir J. 2020; 55:2000688. 10.1183/13993003.00688-2020 32269089 PMC7144263

[14] Muus C, Luecken MD, Eraslan G, Waghray A, Heimberg G, Sikkema L, Kobayashi Y, Vaishnav ED, Subramanian A, Smilie C, Jagadeesh K, Duong ET, Fiskin E, et al Integrated analyses of single-cell atlases reveal age, gender, and smoking status associations with cell type-specific expression of mediators of SARS-CoV-2 viral entry and highlights inflammatory programs in putative target cells. 2020 10.1101/2020.04.19.049254

[15] Kovalchuk O, Kovalchuk I. Cannabinoids as anticancer therapeutic agents. Cell Cycle. 2020; 19:961–89. 10.1080/15384101.2020.1742952 32249682 PMC7217364

[16] Fitzcharles MA, Clauw DJ, Hauser W. A cautious hope for cannabidiol (CBD) in rheumatology care. Arthritis Care Res (Hoboken). 2020. [Epub ahead of print]. 10.1002/acr.24176 32144889

[17] Hoffmann M, Kleine-Weber H, Schroeder S, Krüger N, Herrler T, Erichsen S, Schiergens TS, Herrler G, Wu NH, Nitsche A, Müller MA, Drosten C, Pöhlmann S. SARS-CoV-2 cell entry depends on ACE2 and TMPRSS2 and is blocked by a clinically proven protease inhibitor. Cell. 2020; 181:271–80.e8. 10.1016/j.cell.2020.02.052 32142651 PMC7102627

[18] Imai Y, Kuba K, Rao S, Huan Y, Guo F, Guan B, Yang P, Sarao R, Wada T, Leong-Poi H, Crackower MA, Fukamizu A, Hui CC, et al. Angiotensin-converting enzyme 2 protects from severe acute lung failure. Nature. 2005; 436:112–16. 10.1038/nature03712 16001071 PMC7094998

[19] Kuba K, Imai Y, Rao S, Gao H, Guo F, Guan B, Huan Y, Yang P, Zhang Y, Deng W, Bao L, Zhang B, Liu G, et al. A crucial role of angiotensin converting enzyme 2 (ACE2) in SARS coronavirus-induced lung injury. Nat Med. 2005; 11:875–79. 10.1038/nm1267 16007097 PMC7095783

[20] Aizpurua-Olaizola O, Soydaner U, Öztürk E, Schibano D, Simsir Y, Navarro P, Etxebarria N, Usobiaga A. Evolution of the cannabinoid and terpene content during the growth of cannabis sativa plants from different chemotypes. J Nat Prod. 2016; 79:324–31. 10.1021/acs.jnatprod.5b00949 26836472

[21] Russo EB. Taming THC: potential cannabis synergy and phytocannabinoid-terpenoid entourage effects. Br J Pharmacol. 2011; 163:1344–64. 10.1111/j.1476-5381.2011.01238.x 21749363 PMC3165946

[22] Ligresti A, Moriello AS, Starowicz K, Matias I, Pisanti S, De Petrocellis L, Laezza C, Portella G, Bifulco M, Di Marzo V. Antitumor activity of plant cannabinoids with emphasis on the effect of cannabidiol on human breast carcinoma. J Pharmacol Exp Ther. 2006; 318:1375–87. 10.1124/jpet.106.105247 16728591

[23] De Petrocellis L, Ligresti A, Schiano Moriello A, Iappelli M, Verde R, Stott CG, Cristino L, Orlando P, Di Marzo V. non-THC cannabinoids inhibit prostate carcinoma growth in vitro and in vivo: pro-apoptotic effects and underlying mechanisms. Br J Pharmacol. 2013; 168:79–102. 10.1111/j.1476-5381.2012.02027.x 22594963 PMC3570006

[24] Brake SJ, Barnsley K, Lu W, McAlinden KD, Eapen MS, Sohal SS. Smoking upregulates angiotensin-converting enzyme-2 receptor: a potential adhesion site for novel coronavirus SARS-CoV-2 (Covid-19). J Clin Med. 2020; 9:841. 10.3390/jcm9030841 32244852 PMC7141517

[25] Ribeiro A, Almeida VI, Costola-de-Souza C, Ferraz-de-Paula V, Pinheiro ML, Vitoretti LB, Gimenes-Junior JA, Akamine AT, Crippa JA, Tavares-de-Lima W, Palermo-Neto J. Cannabidiol improves lung function and inflammation in mice submitted to LPS-induced acute lung injury. Immunopharmacol Immunotoxicol. 2015; 37:35–41. 10.3109/08923973.2014.976794 25356537

[26] Lamarre S, Frasse P, Zouine M, Labourdette D, Sainderichin E, Hu G, Le Berre-Anton V, Bouzayen M, Maza E. Optimization of an RNA-seq differential gene expression analysis depending on biological replicate number and library size. Front Plant Sci. 2018; 9:108. 10.3389/fpls.2018.00108 29491871 PMC5817962

[27] Shi CS, Nabar NR, Huang NN, Kehrl JH. SARS-coronavirus open reading frame-8b triggers intracellular stress pathways and activates NLRP3 inflammasomes. Cell Death Discov. 2019; 5:101. 10.1038/s41420-019-0181-7 31231549 PMC6549181

[28] Blagosklonny MV. Koschei the immortal and anti-aging drugs. Cell Death Dis. 2014; 5:e1552. 10.1038/cddis.2014.520 25476900 PMC4649836

[29] Franceschi C, Garagnani P, Parini P, Giuliani C, Santoro A. Inflammaging: a new immune-metabolic viewpoint for age-related diseases. Nat Rev Endocrinol. 2018; 14:576–90. 10.1038/s41574-018-0059-4 30046148

[30] Prattichizzo F, Bonafè M, Olivieri F, Franceschi C. Senescence associated macrophages and “macroph-aging”: are they pieces of the same puzzle? Aging (Albany NY). 2016; 8:3159–60. 10.18632/aging.101133 27941213 PMC5270660

[31] Kim D, Langmead B, Salzberg SL. HISAT: a fast spliced aligner with low memory requirements. Nat Methods. 2015; 12:357–60. 10.1038/nmeth.3317 25751142 PMC4655817

[32] Liao Y, Smyth GK, Shi W. featureCounts: an efficient general purpose program for assigning sequence reads to genomic features. Bioinformatics. 2014; 30:923–30. 10.1093/bioinformatics/btt656 24227677

[33] Love MI, Huber W, Anders S. Moderated estimation of fold change and dispersion for RNA-seq data with DESeq2. Genome Biol. 2014; 15:550. 10.1186/s13059-014-0550-8 25516281 PMC4302049

[34] Benjamini Y, Hochberg Y. Controlling the False Discovery Rate: A Practical and Powerful Approach to Multiple Testing. Journal of the Royal Statistical Society Series B (Methodological). 1995; 57:289–300. 10.1111/j.2517-6161.1995.tb02031.x

