# Supplementary material for: In search of preventive strategies: novel high-CBD Cannabis sativa extracts modulate ACE2 expression in COVID-19 gateway tissues
Source: Aging (Albany NY). 2020 Nov 22;12(22):22425–44. doi: 10.18632/aging.202225 (PMC7746344; doi:10.18632/aging.202225)
Supplement: Supplementary Tables [file aging-12-202225-s001..pdf]

SUPPLEMENTARY TABLES

Supplementary Table 1. Correlation analysis between the downregulation of ACE2 and TMPRSS2 and the level of cannabinoids.

**EpiOral#2 (Figures 3, 8)**	**ACE2**	**TMPRSS2**	**THC**	**CBD**	**CBGA**	**CBN**	**TOTAL**
#1	1.59	0.30	0.28	11.00	0.08	0.04	11.40
#7	1.10	0.22	0.35	10.46	0.09	0.05	10.95
#45	0.52	0.67	0.14	7.92	0.04	0.05	8.15
#130	0.80	0.08	0.77	9.04	0.31	0.06	10.18
#131	0.93	0.92	0.27	11.10	0.27	0.03	11.66
#317	0.71	0.32	1.00	21.81	0.62	0.08	23.51
**Correlation**	ACE2		**-0.26**	**-0.11**	**-0.35**	**-0.42**	**-0.13**
		TMPRSS2	**-0.55**	**-0.13**	**-0.14**	**-0.53**	**-0.16**
**EpiOral#1 (Figure 4A)**	**ACE2**		**THC**	**CBD**	**CBGA**	**CBN**	**TOTAL**
#81	1.56		0.63	13.59	0.10	0.06	14.39
#90	1.05		1.55	6.18	0.06	0.18	7.96
#130	1.89		0.77	9.04	0.31	0.06	10.18
#131	0.86		0.27	11.10	0.27	0.03	11.66
Correlation	ACE2		-0.02	0.19	0.29	-0.24	0.22
EpiOral#1 (Figures 4B, 7A)	ACE2	TMPRSS2	THC	CBD	CBGA	CBN	TOTAL
#1	1.51	1.32	0.28	11.00	0.08	0.04	11.40
#7	1.33	1.58	0.35	10.46	0.09	0.05	10.95
#9	1.60	2.01	0.31	10.37	0.31	0.05	11.03
#45	1.81	1.42	0.14	7.92	0.04	0.05	8.15
#115	1.13	1.42	0.39	13.52	0.13	0.09	14.14
#129	1.34	1.34	0.41	11.23	0.38	0.14	12.15
#157	1.37	1.41	0.27	11.10	0.27	0.03	11.66
#167	1.78	1.44	0.27	11.10	0.27	0.03	11.66
#169	1.60	1.24	0.21	6.13	0.14	0.06	6.54
**Correlation**	ACE2		**-0.80**	**-0.63**	**-0.09**	**-0.53**	**-0.64**
		TMPRSS2	**0.17**	**0.18**	**0.27**	**-0.20**	**0.19**
**EpiOral#1 (Figure 4C)**	**ACE2**		**THC**	**CBD**	**CBGA**	**CBN**	**TOTAL**
#1	0.67		0.28	11.00	0.08	0.04	11.40
#7	1.31		0.35	10.46	0.09	0.05	10.95
#9	1.12		0.31	10.37	0.31	0.05	11.03
#45	0.77		0.14	7.92	0.04	0.05	8.15
#115	1.10		0.39	13.52	0.13	0.09	14.14
#129	0.97		0.41	11.23	0.38	0.14	12.15
#157	1.49		0.27	11.10	0.27	0.03	11.66
#167	1.53		0.27	11.10	0.27	0.03	11.66
#169	1.84		0.21	6.13	0.14	0.06	6.54
**Correlation**	ACE2		**-0.11**	**-0.32**	**0.22**	**-0.21**	**-0.30**
**EpiAir#1 (Figure 2)**	**ACE2**		**THC**	**CBD**	**CBGA**	**CBN**	**TOTAL**
#5	1.47		0.38	11.42	0.04	0.03	11.87
#10	1.79		0.38	9.41	0.24	0.06	10.09
#31	1.45		6.21	8.90	0.09	0.05	15.24
#49	1.20		0.09	9.06	0.07	0.04	9.25
#81	0.89		0.63	13.59	0.10	0.06	14.39
#114	1.27		0.18	10.41	0.11	0.07	10.76
#155	1.06		0.27	11.10	0.27	0.03	11.66
#166	0.83		0.27	11.10	0.27	0.03	11.66
#169	1.00		0.21	6.13	0.14	0.06	6.54

#207	0.85		8.93	7.84	0.23	0.08	17.08
**Correlation**	ACE2		**-0.15**	**-0.06**	**-0.27**	**-0.16**	**-0.20**
**EpiIntestinal (Figures 5A, 7B)**	**ACE2**	**TMPRSS2**	**THC**	**CBD**	**CBGA**	**CBN**	**TOTAL**
#1	0.95	1.20	0.28	11.00	0.08	0.04	11.40
#7	0.88	1.28	0.35	10.46	0.09	0.05	10.95
#9	1.02	1.27	0.31	10.37	0.31	0.05	11.03
#45	2.35	1.44	0.14	7.92	0.04	0.05	8.15
#115	0.88	1.28	0.39	13.52	0.13	0.09	14.14
#129	1.17	1.43	0.41	11.23	0.38	0.14	12.15
#130	1.24	1.48	0.77	9.04	0.31	0.06	10.18
#167	0.97	1.51	0.27	11.10	0.27	0.03	11.66
#274	0.88	1.29	0.30	13.93	0.38	0.04	14.64
**Correlation**	ACE2		**-0.27**	**-0.72**	**-0.35**	**-0.04**	**-0.75**
		TMPRSS2	**0.26**	**-0.44**	**0.30**	**0.15**	**-0.38**
**EpiIntestinal (Figure 5B)**	**ACE2**		**THC**	**CBD**	**CBGA**	**CBN**	**TOTAL**
#1	1.41		0.28	11.00	0.08	0.04	11.40
#7	1.19		0.35	10.46	0.09	0.05	10.95
#9	0.92		0.31	10.37	0.31	0.05	11.03
#45	1.92		0.14	7.92	0.04	0.05	8.15
#115	1.02		0.39	13.52	0.13	0.09	14.14
#129	1.23		0.41	11.23	0.38	0.14	12.15
#130	1.00		0.77	9.04	0.31	0.06	10.18
#167	1.15		0.27	11.10	0.27	0.03	11.66
#274	1.02		0.30	13.93	0.38	0.04	14.64
**Correlation**	ACE2		**-0.54**	**-0.56**	**-0.63**	**-0.11**	**-0.64**
**EpiAirwayFT#2 (Figures 6A, 9A)**	**ACE2**	**TMPRSS2**	**THC**	**CBD**	**CBGA**	**CBN**	**TOTAL**
#1	6.74	0.88	0.28	11.00	0.08	0.04	11.40
#5	3.28	0.88	0.38	11.42	0.04	0.03	11.87
#7	4.27	0.81	0.35	10.46	0.09	0.05	10.95
#10	3.20	0.95	0.38	9.41	0.24	0.06	10.09
#81	1.45	0.73	0.63	13.59	0.10	0.06	14.39
#129	3.37	0.76	0.41	11.23	0.38	0.14	12.15
#169	5.57	0.83	0.21	6.13	0.14	0.06	6.54
#317	7.06	0.93	1.00	21.81	0.62	0.08	23.51
**Correlation**	ACE2		0.14	0.28	0.38	-0.12	0.28
		TMPRSS2	**0.15**	**0.22**	**0.30**	**-0.37**	**0.22**
**EpiAirway#3 (Figures 6B, 9B)**	**ACE2**	**TMPRSS2**	**THC**	**CBD**	**CBGA**	**CBN**	**TOTAL**
#1	4.12	0.88	0.28	11.00	0.08	0.04	11.40
#5	10.00	0.88	0.38	11.42	0.04	0.03	11.87
#7	4.12	0.81	0.35	10.46	0.09	0.05	10.95
#10	2.33	0.63	0.38	9.41	0.24	0.06	10.09
#45	2.33	0.70	0.14	7.92	0.04	0.05	8.15
#81	4.12	0.55	0.63	13.59	0.10	0.06	14.39
#129	3.89	0.55	0.41	11.23	0.38	0.14	12.15
#317	5.83	0.58	1.00	21.81	0.62	0.08	23.51
**Correlation**	ACE2		**0.27**	**0.34**	**-0.02**	**-0.22**	**0.32**
		TMPRSS2	**-0.52**	**-0.37**	**-0.63**	**-0.75**	**-0.39**

First column shows the experiment (with the reference to a corresponding Figure). Data in “ACE2” and “TMPRSS2” columns show the fold downregulation of their expression. “THC”, “CBD”, “CBGA”, “CBN” and “Total” columns show the molar concentrations for each extract. “Correlation” shows correlation between the fold downregulation and the concentration of cannabinoids for each experiment.

Supplementary Table 2. Correlation analysis between the downregulation of ACE2 and TMPRSS2 and the level of terpenes.

**EpiOral (Figures 3, 8)**	**ACE2**	**TMPRSS2**	**Terpenes**
#1	1.59	0.3	12.34
#7	1.1	0.22	6.95
#317	0.71	0.32	1.93
Correlation	ACE2		**0.99**
		TMPRSS2	**-0.17**
**EpiOral (Figures 4B, 7A)**	**ACE2**	**TMPRSS2**	**Terpenes**
#1	1.51	1.32	12.34
#7	1.33	1.58	6.95
#9	1.60	2.01	14.02
Correlation	ACE2		**0.99**
		TMPRSS2	**0.35**
**EpiOral (Figure 4C)**	**ACE2**	**TMPRSS2**	**Terpenes**
#1	1.35		12.34
#7	1.11		6.95
#9	0.79		14.02
Correlation	ACE2		**-0.31**
**EpiIntestinal (Figures 5A, 7B)**	ACE2	TMPRSS2	Terpenes
#1	0.95	1.20	12.34
#7	0.88	1.28	6.95
#9	1.02	1.27	14.02
Correlation	ACE2		**0.97**
		TMPRSS2	**-0.35**
**EpiIntestinal (Figure 5B)**	**ACE2**	**TMPRSS2**	**Terpenes**
#1	1.41		12.34
#7	1.19		6.95
#9	0.92		14.02
Correlation	ACE2		**-0.29**
**EpiAir (Figures 6A, 9A)**	**ACE2**	**TMPRSS2**	**Terpenes**
#1	6.74		12.34
#7	4.27		6.95
#81	1.45		4.55
#317	7.06		1.93
Correlation	ACE2		**0.21**
Correlation	TMPRSS2		**0.02**

First column shows the experiment (with the reference to a Figure). Data in “ACE2” and “TMPRSS2” columns show the fold downregulation of their expression. “Terpenes” column shows the percentage of all terpenes in each extract. “Correlation” shows correlation between the fold downregulation and the concentration of terpenes in each extract.

Supplementary Table 3. Comparison of fold downregulation in ACE2 expression in different tissues and cells.

Cultivars	Oral#1	Oral#2	Air#1	AirFT#2	Air#3	Intest	Average
#1	1.09	1.59		6.74	4.12	1.18	**2.94**
#5			1.47	3.28	10		**4.92**
#7	1.32	1.1		4.27	4.12	1.02	**2.37**
#9	1.36					0.98	**1.17**
#10			1.79	3.2	2.33		**2.44**
#31			1.45				**1.45**
#45	1.26	0.52			2.33	2.13	**1.56**
#49			1.2				**1.20**
#81	1.56		0.89	1.45	4.12		**2.01**
#90	1.05						**1.05**
#114			1.27				**1.27**
#115	1.12					0.95	**1.04**
#129	1.18			3.37	3.89	1.2	**2.41**
#130	1.89	0.8				1.12	**1.27**
#131	0.86	0.93					**0.90**
#155			1.06				**1.06**
#157	1.43						**1.43**
#166	1.55		0.83				**1.19**
#167	1.65					1.07	**1.36**
#169	1.72		1	5.57			**2.76**
#207			0.85	0.85			**0.85**
#274						0.95	**0.95**
#317		0.71		7.06	5.83		**4.53**
**Average**	**1.36**	**0.94**	**1.18**	**3.98**	**4.59**	**1.18**	**1.83**
